# Effects of continuous care based on a mobile health platform on self-management behaviors and quality of life in patients with acute leukemia

**DOI:** 10.3389/fmed.2026.1880270

**Published:** 2026-07-22

**Authors:** Wulipaxi Habasi, Ayinaer Shaheba, Weifang Xu, Hui Zhang

**Affiliations:** Disinfection and Distribution Center, The First Affiliated Hospital of Xinjiang Medical University, Urumqi, Xinjiang, China

**Keywords:** acute leukemia, cancer-related fatigue, continuous nursing, Epworth Sleepiness Scale, mobile health, quality of life, self-management

## Abstract

**Aim:**

To evaluate the effects of continuous care delivered via a mobile health (mHealth) platform on self-management behaviors, quality of life (QoL), medication adherence, psychological distress, daytime sleepiness, cancer-related fatigue (CRF), complications, and rehospitalization in patients with acute leukemia (AL).

**Methods:**

A total of 108 AL patients discharged between January 2024 and January 2025 were randomly assigned to an observation group (*n* = 54) receiving mHealth-based continuous care plus routine follow-up, or a control group (*n* = 54) receiving routine continuous care only. The intervention lasted 6 months. Seven patient-reported outcome scales were assessed at baseline, 3 months, and 6 months: self-management behavior (SUPPH with three subscales), QoL (EORTC QLQ-C30 global health status and five functional subscales), medication adherence (MMAS-8), anxiety (SAS), depression (SDS), daytime sleepiness (ESS), cancer-related fatigue (PFS with four subscales). Complications and rehospitalization were recorded throughout the 6-month period. Complications and unplanned rehospitalization were prospectively recorded as cumulative endpoints throughout the 6-month intervention period. For repeated measures, two-way repeated measures ANOVA (group × time) was applied.

**Results:**

Significant group × time interactions were observed for all continuous variables (all *p* < 0.001). At 3 and 6 months, the observation group showed progressively better scores. For example, SUPPH total score at 6 months: 86.42 ± 6.13 vs. 71.25 ± 8.54 (*p* < 0.001); QLQ-C30 global health status: 78.36 ± 7.21 vs. 62.45 ± 9.18 (*p* < 0.001); MMAS-8: 7.18 ± 0.75 vs. 6.05 ± 1.21 (*p* < 0.001); SAS: 45.21 ± 4.13 vs. 50.36 ± 5.02 (*p* < 0.001); SDS: 46.38 ± 5.22 vs. 53.19 ± 5.64 (*p* < 0.001); ESS: 7.32 ± 2.15 vs. 11.68 ± 3.04 (*p* < 0.001); PFS total: 3.25 ± 1.02 vs. 5.73 ± 1.48 (*p* < 0.001). All subscales followed similar patterns. Complication rate (11.11% vs. 29.63%, *p* = 0.016) and rehospitalization rate (7.41% vs. 24.07%, *p* = 0.012) were lower in the observation group.

**Conclusion:**

Continuous care based on an mHealth platform significantly improves self-management, QoL, medication adherence, psychological outcomes, daytime sleepiness, and fatigue at 3 and 6 months, and reduces complications and rehospitalizations in this single-center cohort of AL patients. These findings require multicenter validation in more diverse populations.

## Introduction

Acute leukemia (AL) is a heterogeneous group of hematologic malignancies characterized by rapid proliferation of immature hematopoietic cells (1). Although induction chemotherapy can achieve complete remission in a substantial proportion of patients, the disease course is often protracted, requiring repeated hospitalizations for consolidation or maintenance therapy, as well as intensive supportive care (2, 3). Between hospital stays, patients face numerous challenges including myelosuppression-related infections, bleeding tendencies, chemotherapy-induced nausea and vomiting, nutritional deterioration, and psychological distress (4). Effective self-management—monitoring vital signs, recognizing early signs of infection, adhering to complex medication schedules—is crucial for reducing adverse events (5). Conventional continuous care models rely on telephone follow-up, outpatient visits, and printed materials. These approaches have inherent limitations: intermittent contact may miss early warning signs, and one-way communication does not allow real-time problem-solving, leaving patients feeling isolated (6). Moreover, AL patients can deteriorate rapidly (e.g., febrile neutropenia), and delayed recognition may lead to severe sepsis or death (7).

Mobile health (mHealth) technologies can bridge this post-discharge gap (8). Using smartphones and WeChat mini-programs, providers can deliver educational content, collect patient-reported data daily, send medication reminders, and enable two-way communication (9). While mHealth interventions have improved self-efficacy and QoL in oncology (10), evidence specific to AL is limited, and few randomized trials have simultaneously measured multiple clinical endpoints.

Therefore, this study aimed to evaluate the effects of an mHealth platform-based continuous care program on self-management behaviors, QoL, medication adherence, anxiety/depression, daytime sleepiness, cancer-related fatigue, complications, and rehospitalization in AL patients, compared with routine care.

## Methods

### Study design and setting

This was a single-center, parallel-group, randomized controlled trial conducted at our hospital between January 2024 and January 2025. The trial was registered in the Chinese Clinical Trial Registry (ChiCTR2300071133). The study was approved by the hospital ethics committee. All participants provided written informed consent.

### Sample size calculation

Based on a pilot study (*n* = 20/group), the mean SUPPH total score in the control group was 68.5 ± 10.2, and a clinically meaningful difference of 8 points (Cohen’s d ≈ 0.5) was targeted. At *α* = 0.05 and 80% power, 26 participants per group were needed. Accounting for a 20% dropout rate, the minimum required sample size was 33 participants per group. Given the prespecified 12-month recruitment window and continuous patient enrollment, we ultimately included 54 patients per group (total *n* = 108), which further improved statistical power for secondary outcome analyses.

### Participants

Inclusion criteria: (1) diagnosis of acute leukemia (AML or ALL) according to the revised WHO criteria; (2) age ≥18 years; (3) achieved complete remission (CR) or partial remission (PR) after induction chemotherapy and clinically stable for discharge; (4) owned a smartphone and could operate WeChat or a mobile application; (5) willing to participate and provide informed consent.

Exclusion criteria: (1) severe heart, liver, or renal failure; (2) diagnosed mental illness or cognitive impairment; (3) life expectancy <6 months; (4) unable to complete follow-up.

### Randomization and blinding

Of 127 screened patients, 108 were randomized 1:1 using a computer-generated sequence with sealed opaque envelopes. Due to the nature of the intervention, participants and intervention nurses could not be blinded, but outcome assessors and data analysts were blinded throughout.

### Conceptual framework

The mHealth platform served as a central coordinating infrastructure integrating four functional layers: (1) real-time data collection via a daily health diary, transforming episodic reports into continuous data streams; (2) automated triage and alerting based on prespecified thresholds (e.g., fever ≥38.5 °C), enabling a “30-min callback” promise; (3) asynchronous multi-channel communication and on-demand video consultation; and (4) educational push notifications with quizzes and a moderated peer support forum. These layers collectively restructured care from passive, scheduled follow-up to proactive, risk-stratified surveillance (Supplementary Figure S1).

### Interventions

#### Control group (routine continuous care)

Before discharge, patients received a printed health education booklet, a 30-min face-to-face discharge instruction, telephone follow-ups at 1 and 3 months, and outpatient clinic visits at 3 and 6 months.

#### Observation group (mHealth-based continuous care)

In addition to the above routine care, patients used the “Leukemia Home Care Assistant” WeChat mini-program for 6 months. The intervention components described below were operationalized, monitored, and coordinated through the mHealth platform, which served as the technological infrastructure for the integrated workflow described in the Conceptual Framework.

##### Daily health diary

Patients were required to complete a 2-min diary every morning (8:00–10:00) recording: body temperature, systolic/diastolic blood pressure, presence of petechiae or abnormal bleeding (yes/no), fatigue level (0–10 numeric rating scale), appetite (1 = poor, 2 = fair, 3 = good), stool color and consistency (normal/black/tarry/diarrhea). The platform stored all diary entries chronologically, allowing nurses to view trends over time and enabling patients to track their own progress. The system automatically flagged abnormal entries: fever ≥38.5 °C, black/tarry stools, systolic BP < 90 mmHg, or fatigue ≥8. These triggered a red alert instantly sent to the on-duty nurse’s mobile phone, prompting a callback within 30 min.

##### Medication reminder and adherence log

Twice daily (08:00 and 20:00) automatic push notifications reminded patients to take oral chemotherapy (mercaptopurine, methotrexate), antibiotics (levofloxacin, cefixime), antifungals (fluconazole, posaconazole), and G-CSF injections (if prescribed). Patients clicked “taken” or “snooze” (20 min). Missed doses without confirmation for >2 h were flagged on the nurse dashboard, and the nurse called the patient for follow-up.

##### Educational push notifications

Every Monday and Thursday, short (3–5 min) videos or infographics were sent. Topics included: (1) how to take temperature and recognize fever, (2) seven steps of hand hygiene, (3) nutritional recipes for neutropenic diet, (4) managing oral mucositis with saline/baking soda rinses, (5) psychological coping – relaxation breathing, (6) emergency signs requiring immediate medical attention (e.g., chills, bleeding gums, confusion). Content was curated and updated by the nursing team through a platform-admin interface. Each message was followed by a 2-question quiz; correct answers reinforced learning, and incorrect answers prompted automatic re-delivery of the educational material. Nurses could view knowledge gap statistics on the dashboard to deliver targeted supplementary teaching.

##### Online consultation

A dedicated hematology nurse was available weekdays 08:00–18:00 (response within 4 h) and weekends/holidays 09:00–12:00 (response within 12 h). Patients could send text, voice messages, or photos (e.g., skin rashes, oral ulcers) via the mini-program. The platform provided a unified inbox where all patient queries were queued, categorized, and triaged. If the nurse deemed it necessary, a hematologist or psychologist (video call scheduled within 24 h) was consulted. Psychological video sessions were offered bi-weekly for patients with SAS ≥ 50 or SDS ≥ 53, with referrals automatically triggered by platform-collected outcome data.

##### Peer support group

An anonymized, moderated discussion forum allowed patients to share experiences (e.g., coping with fatigue, managing taste changes). The platform ensured anonymity through user ID masking and provided moderation tools (keyword filtering, flagged content review) to maintain a safe environment. A nurse posted weekly guided questions to encourage interaction.

##### Compliance monitoring

Nurses checked the platform dashboard every morning (including weekends). Any patient who did not complete the daily diary by 10:30 received an in-app reminder; if still missing by 12:00, a phone call was made. Adherence to diary completion was >92% in the observation group based on weekly platform reports.

Collectively, the above components operated synergistically through the mHealth platform to transform the traditional episodic, passive follow-up model into continuous, proactive, risk-stratified care. The integrated workflow of automated monitoring, tiered alerting, targeted education, and on-demand support addressed both the physical and psychological needs of AL patients during home-based myelosuppression periods, which cannot be fully achieved via routine telephone follow-up alone.

### Outcome measures

Repeated patient-reported assessments were performed at baseline (discharge day), 3 months post-discharge, and 6 months post-discharge (end of intervention). Complications and unplanned rehospitalizations were continuously and prospectively documented throughout the 6-month period. The same instruments and subscales as previously described were used.

### Primary outcome

#### Self-management behavior

Chinese version of the Strategies Used by People to Promote Health (SUPPH) questionnaire (11). It contains 25 items rated on a 5-point Likert scale (1 = never to 5 = always), with total scores ranging from 25 to 125. Higher scores indicate better self-management behaviors. The scale includes three subscales: self-determination (10 items), positive attitude (9 items), and self-stress reduction (6 items).

### Secondary outcomes

#### Quality of life (QoL)

The European Organisation for Research and Treatment of Cancer Quality of Life Questionnaire Core 30 (EORTC QLQ-C30) version 3.0. The original instrument was developed by Aaronson et al. (12), and the validated simplified Chinese version used in this study was confirmed by Wan et al. (13). This study used the Global Health Status/QoL scale (items 29 and 30), which is linearly transformed to a 0–100 score. Higher scores represent better overall QoL. Additionally, the functional summary score (physical, role, emotional, cognitive, social functioning) was recorded (each 0–100).

#### Medication adherence

The Morisky Medication Adherence Scale-8 (MMAS-8). The original 8-item scale was developed by Morisky et al. (14), and the validated Chinese version was provided by Shi et al. (15). Scores range from 0 to 8. Scores ≥6 indicate adequate adherence, 4–5 moderate adherence, and <4 low adherence.

#### Anxiety and depression

The Zung Self-Rating Anxiety Scale (SAS) and the Zung Self-Rating Depression Scale (SDS). The original scales were developed by Zung (16, 17), and validated Chinese versions have been widely utilized in Chinese clinical populations (18). Standardized scores range from 20 to 80 (20 points added to raw score). A cutoff of SAS score ≥50 indicates clinically significant anxiety. A cutoff of SDS score ≥53 indicates clinically significant depression.

#### Daytime sleepiness

The Epworth Sleepiness Scale (ESS), originally developed by Johns (19) and validated in Chinese by Chen et al. (20). It is an 8-item self-administered questionnaire assessing the likelihood of dozing off in eight common sedentary situations. Each item is scored from 0 (never doze) to 3 (high chance of dozing). Total score ranges from 0 to 24. A score ≤7 is considered normal, 8–9 borderline, 10–15 moderate excessive daytime sleepiness, and ≥16 severe sleepiness.

#### Cancer-related fatigue

The Piper Fatigue Scale (PFS) – revised version (21) and its validated Chinese version (22). This multidimensional instrument consists of 22 items across four dimensions: (1) behavioral/severity (6 items), (2) affective meaning (5 items), (3) sensory (5 items), and (4) cognitive/mood (6 items). Each item is rated on a 0–10 numeric rating scale. Total PFS score is the mean of all 22 items, ranging from 0 to 10. Higher scores indicate greater fatigue severity. A score of 0 indicates no fatigue, 1–3 mild fatigue, 4–6 moderate fatigue, and 7–10 severe fatigue.

#### Complication rate

Any documented event during the 6 months, including infection (fever ≥38.5 °C with neutropenia, pneumonia, sepsis), bleeding (epistaxis, gastrointestinal bleeding, intracranial hemorrhage), oral mucositis (Grade ≥2 by WHO criteria), and other chemotherapy-related toxicities.

#### Rehospitalization rate

Unplanned hospital admission due to disease progression, severe complication, or any cause requiring inpatient care (excluding routine chemotherapy courses scheduled in advance).

### Prespecified analysis plan

All analyses were conducted according to the intention-to-treat (ITT) principle, with participants analyzed in the groups to which they were originally randomized. Baseline characteristics were compared using independent t-tests and chi-square tests. The primary outcome was analyzed via independent t-test at 6 months and two-way repeated measures ANOVA (Group × Time) across three time points. Secondary outcomes followed the same analytical framework. Missing data (<5%) would be handled via multiple imputation; no dropouts occurred in this study, so imputation was not required. The Benjamini-Hochberg false discovery rate (FDR) method was applied to control for Type I error inflation across secondary outcomes.

### Statistical analysis

**Figure 1:**
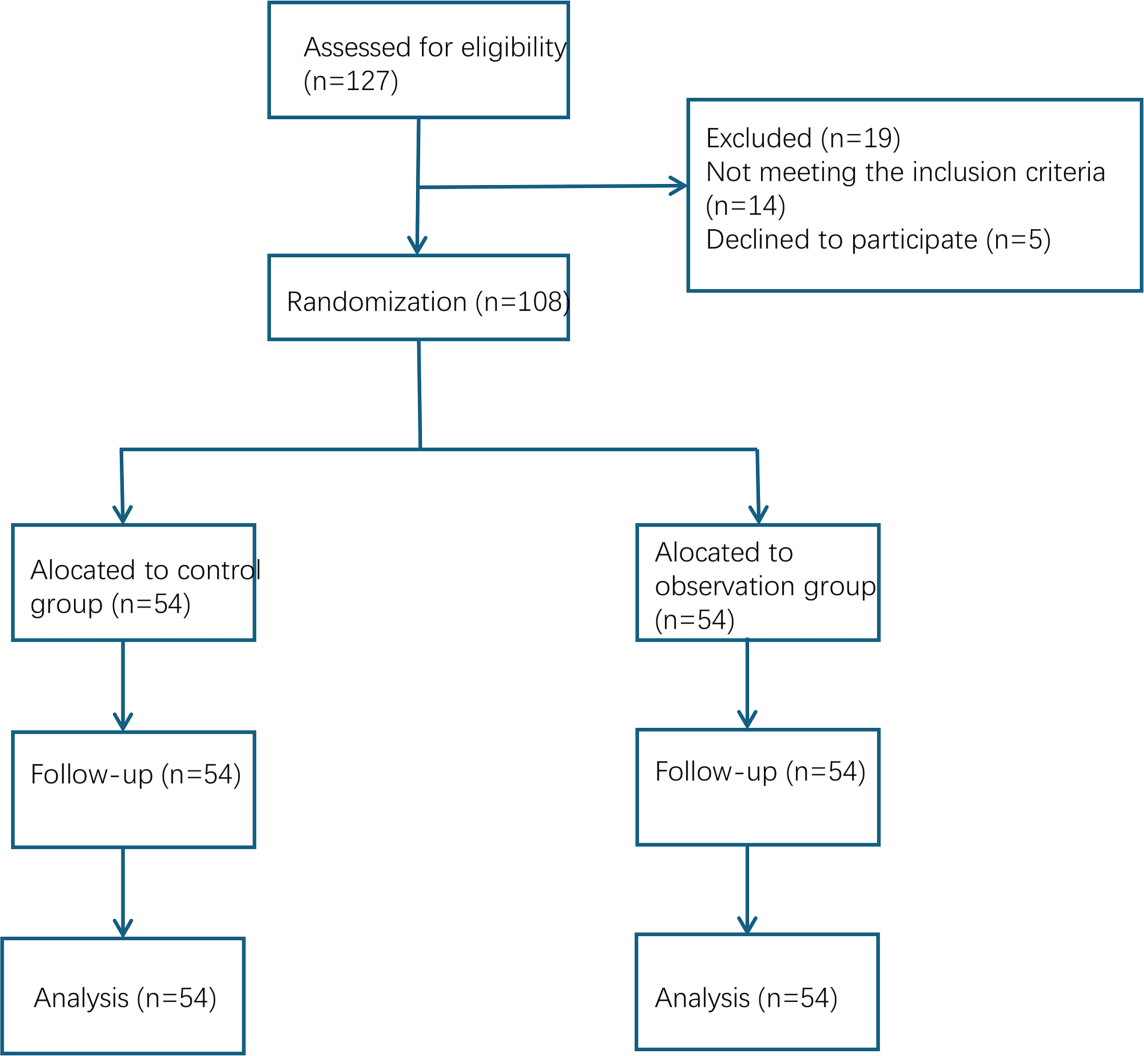
Patient flow chart.

Statistical analysis was performed using SPSS 26.0. Continuous variables are presented as mean ± SD. The Shapiro–Wilk test was first employed to assess normality of data distribution. Normally distributed data were analyzed using the unpaired Student’s t-test; non-normally distributed data were analyzed using the Mann–Whitney U test. A two-way repeated measures ANOVA (factors: Group and Time) with Group × Time interaction was applied for longitudinal comparisons. Post-hoc pairwise comparisons with Bonferroni correction were performed for significant interactions. Categorical variables were compared using chi-square test or Fisher’s exact test. All statistical tests were two-sided, with significance set at *p* < 0.05. Partial η^2^ is reported as effect size for repeated measures ANOVA (see Figure 1).

## Results

### Baseline characteristics

A total of 108 patients completed the 6-month follow-up with no dropouts. Baseline demographic and clinical characteristics were well balanced between the observation and control groups (Table 1). AML- and ALL-stratified baseline characteristics are provided in Supplementary Table S1.

**Table 1:** Baseline characteristics of the two groups.

Characteristic	Observation (*n* = 54)	Control (*n* = 54)	*p*-value
Age (years), mean ± SD	46.3 ± 12.7	47.1 ± 13.2	0.748
Age ≥60 years, *n* (%)	9 (16.7)	11 (20.4)	0.615
Sex, *n* (%)			0.698
Male	31 (57.4)	29 (53.7)	
Female	23 (42.6)	25 (46.3)	
Leukemia subtype, *n* (%)			0.671
AML	38 (70.4)	40 (74.1)	
ALL	16 (29.6)	14 (25.9)	
KPS score at discharge, mean ± SD	72.5 ± 8.6	71.9 ± 9.1	0.728
WBC count at diagnosis (×10^9^/L), mean ± SD	28.6 ± 18.4	30.1 ± 20.2	0.685
Hemoglobin at diagnosis (g/L), mean ± SD	89.4 ± 15.2	87.6 ± 16.8	0.558
Platelet count at diagnosis (×10^9^/L), mean ± SD	52.3 ± 28.6	55.1 ± 30.2	0.618
Induction chemotherapy regimen, *n* (%)			0.632
Standard 7 + 3 (AML) / Hyper-CVAD (ALL)	42 (77.8)	44 (81.5)	
Other regimens	12 (22.2)	10 (18.5)	
Cytogenetic risk category, *n* (%)			0.892
Favorable	10 (18.5)	12 (22.2)	
Intermediate	28 (51.9)	26 (48.1)	
Adverse	16 (29.6)	16 (29.6)	
Received allo-HSCT, *n* (%)	12 (22.2)	14 (25.9)	0.650
Comorbidities, *n* (%)			
Hypertension	8 (14.8)	7 (13.0)	0.781
Diabetes	5 (9.3)	6 (11.1)	0.751
Education level, *n* (%)			0.884
Primary school or below	12 (22.2)	14 (25.9)	
Secondary school	26 (48.1)	24 (44.4)	
College or above	16 (29.6)	16 (29.6)	
Marital status, *n* (%)			0.650
Married	42 (77.8)	40 (74.1)	
Unmarried/divorced/widowed	12 (22.2)	14 (25.9)	
Residence, *n* (%)			0.697
Urban	30 (55.6)	28 (51.9)	
Rural	24 (44.4)	26 (48.1)	

### Primary outcome: self-management behavior

**Figure 2:**
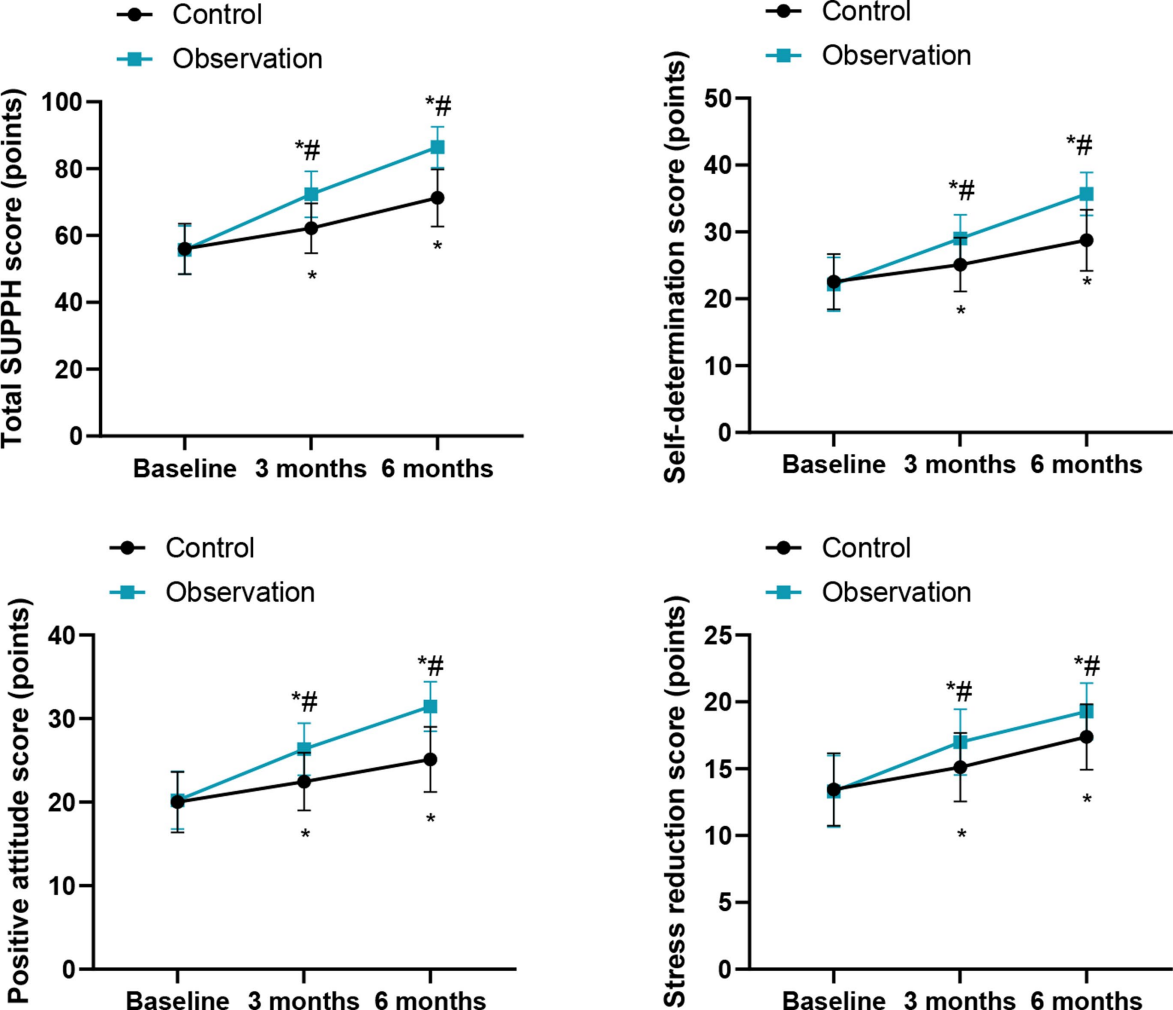
Self-management behavior between the two groups. ^*^p < 0.05, compared with baseline; ^#^*p* < 0.05, compared with control group.

The observation group demonstrated substantially greater improvements in self-management behavior over time compared with the control group, as indicated by a significant group × time interaction for the SUPPH total score (*F*(1.6,169.6) = 52.34, *p* < 0.001, partial η^2^ = 0.331; Table 2; Figure 2). Between-group differences were significant at both 3 and 6 months (*p* < 0.001 for both). All three SUPPH subscales—self-determination, positive attitude, and stress reduction—followed similar patterns, with significantly greater improvements in the observation group at all follow-up time points (*p* < 0.01 for all comparisons; Table 2; Figure 2).

**Table 2:** Summary of continuous outcomes at baseline, 3 months, and 6 months.

Outcome	Group	Baseline	3 months	6 months	Group × Time F (η^2^)	*p*-value
SUPPH total	Observation	55.68 ± 7.23	72.34 ± 6.89*	86.42 ± 6.13*#	52.34 (0.331)	<0.001
	Control	56.02 ± 7.55	62.18 ± 7.45*	71.25 ± 8.54*		
SUPPH-self determination	Observation	22.15 ± 4.02	29.01 ± 3.56*	35.68 ± 3.21*#	44.78 (0.297)	<0.001
	Control	22.56 ± 4.11	25.12 ± 4.01*	28.75 ± 4.56*		
SUPPH-positive attitude	Observation	20.22 ± 3.45	26.34 ± 3.12*	31.45 ± 2.98*#	48.23 (0.313)	<0.001
	Control	20.01 ± 3.62	22.45 ± 3.45*	25.12 ± 3.89*		
SUPPH-stress reduction	Observation	13.31 ± 2.67	16.99 ± 2.45*	19.29 ± 2.11*#	36.78 (0.258)	<0.001
	Control	13.45 ± 2.71	15.11 ± 2.56*	17.38 ± 2.45*		
QLQ-C30 global health	Observation	58.43 ± 9.25	70.12 ± 8.34*	78.36 ± 7.21*#	48.76 (0.315)	<0.001
	Control	59.12 ± 9.56	60.23 ± 9.45	62.45 ± 9.18*		
QLQ-C30 physical	Observation	62.35 ± 10.12	71.56 ± 9.23*	79.88 ± 8.45*#	45.12 (0.299)	<0.001
	Control	61.98 ± 10.56	64.02 ± 10.11	66.23 ± 9.87*		
QLQ-C30 role	Observation	55.67 ± 12.34	66.23 ± 10.45*	74.56 ± 9.12*#	41.23 (0.280)	<0.001
	Control	56.12 ± 11.98	58.44 ± 11.02	60.34 ± 10.45		
QLQ-C30 emotional	Observation	60.22 ± 10.45	69.87 ± 9.12*	76.89 ± 8.22*#	54.67 (0.340)	<0.001
	Control	59.87 ± 10.67	61.34 ± 10.02	63.45 ± 9.88		
QLQ-C30 cognitive	Observation	68.45 ± 9.87	76.12 ± 8.56*	82.34 ± 7.56*#	43.89 (0.293)	<0.001
	Control	67.98 ± 10.01	69.45 ± 9.34	71.22 ± 8.99*		
QLQ-C30 social	Observation	58.99 ± 11.23	69.45 ± 9.56*	77.45 ± 8.67*#	47.23 (0.308)	<0.001
	Control	59.23 ± 10.98	61.02 ± 10.11	63.89 ± 9.56*		
MMAS-8	Observation	5.43 ± 1.02	6.45 ± 0.89*	7.18 ± 0.75*#	38.21 (0.265)	<0.001
	Control	5.58 ± 1.10	5.78 ± 1.05	6.05 ± 1.21*		
SAS	Observation	52.34 ± 5.67	48.12 ± 4.56*	45.21 ± 4.13*#	45.02 (0.298)	<0.001
	Control	51.89 ± 5.45	51.23 ± 5.12	50.36 ± 5.02		
SDS	Observation	55.62 ± 6.01	50.23 ± 5.34*	46.38 ± 5.22*#	47.33 (0.309)	<0.001
	Control	55.01 ± 5.89	54.89 ± 5.76	53.19 ± 5.64*		
ESS	Observation	12.15 ± 2.86	9.23 ± 2.45*	7.32 ± 2.15*#	59.87 (0.361)	<0.001
	Control	11.98 ± 2.94	11.89 ± 3.01	11.68 ± 3.04		
PFS total	Observation	5.82 ± 1.35	4.45 ± 1.18*	3.25 ± 1.02*#	67.12 (0.388)	<0.001
	Control	5.76 ± 1.41	5.70 ± 1.45	5.73 ± 1.48		
PFS-behavioral/severity	Observation	5.95 ± 1.42	4.38 ± 1.22*	3.12 ± 1.08*#	61.45 (0.367)	<0.001
	Control	5.88 ± 1.39	5.91 ± 1.48	5.89 ± 1.52		
PFS-affective meaning	Observation	5.71 ± 1.38	4.23 ± 1.15*	3.01 ± 1.11*#	55.78 (0.345)	<0.001
	Control	5.65 ± 1.44	5.60 ± 1.42	5.62 ± 1.47		
PFS-sensory	Observation	6.01 ± 1.45	4.56 ± 1.28*	3.45 ± 1.15*#	68.34 (0.392)	<0.001
	Control	5.95 ± 1.48	5.93 ± 1.51	5.91 ± 1.38		
PFS-cognitive/mood	Observation	5.62 ± 1.36	4.31 ± 1.20*	3.38 ± 1.05*#	52.67 (0.332)	<0.001
	Control	5.58 ± 1.40	5.55 ± 1.46	5.51 ± 1.44		

*p < 0.05 vs. baseline within group; #*p* < 0.05 vs. 3 months within group. All between-group comparisons at 3 and 6 months were significant at *p* < 0.05 unless otherwise noted. SUPPH, Strategies Used by People to Promote Health; QLQ-C30, Quality of Life Questionnaire Core 30; MMAS-8, Morisky Medication Adherence Scale-8; SAS, Self-Rating Anxiety Scale; SDS, Self-Rating Depression Scale; ESS, Epworth Sleepiness Scale; PFS, Piper Fatigue Scale.

### Secondary outcomes

Significant group × time interactions were observed for all secondary outcomes (all *p* < 0.001), with effect sizes (partial η^2^) ranging from 0.265 to 0.392 (Table 2). At both 3 and 6 months, the observation group scored significantly better than the control group on every measure. After Benjamini-Hochberg false discovery rate correction for multiple secondary endpoints, all between-group differences remained statistically significant (adjusted *p* < 0.05).

**Figure 3:**
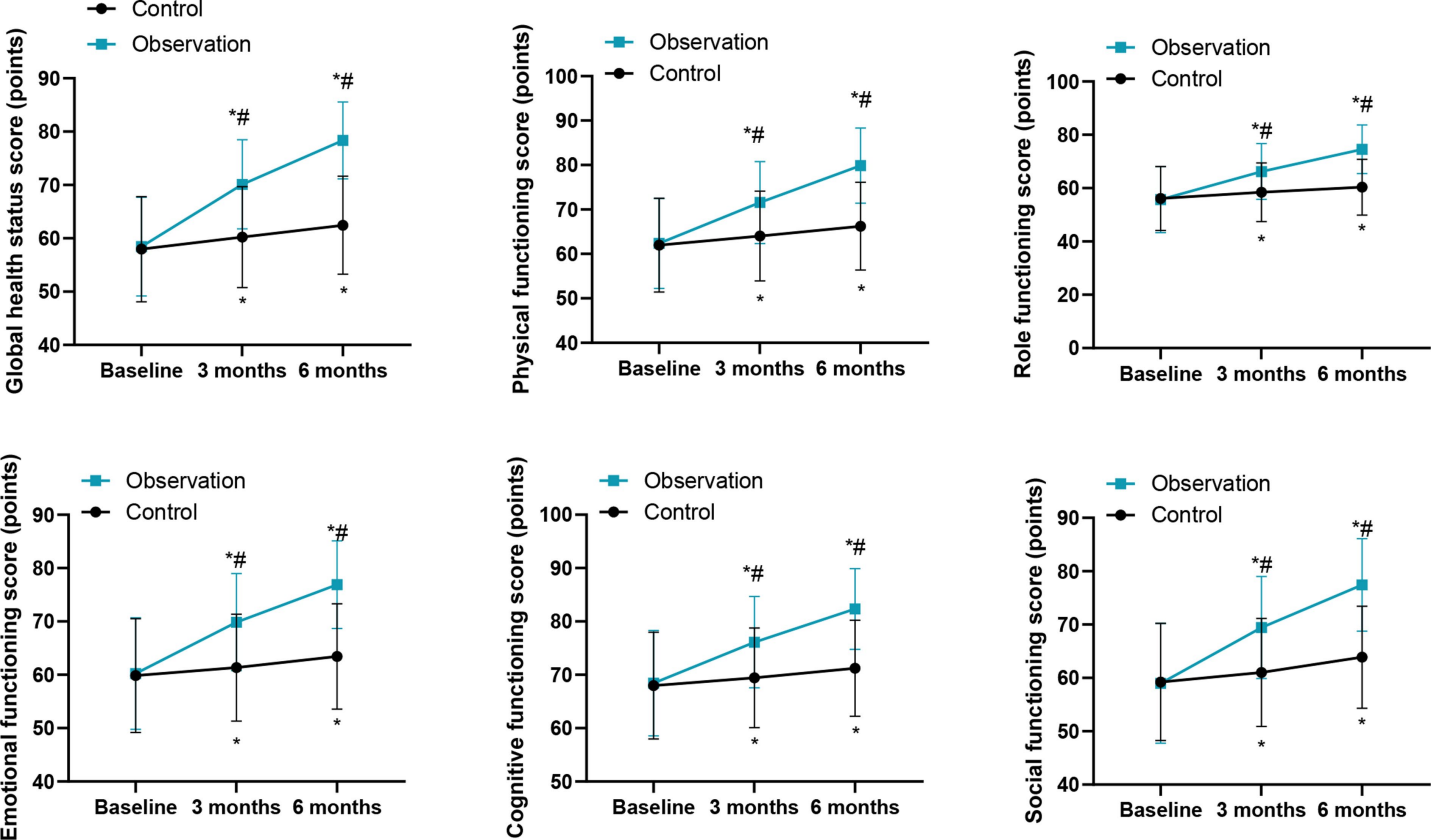
Quality of life between the two groups. ^*^p < 0.05, compared with baseline; ^#^*p* < 0.05, compared with control group.

For quality of life, EORTC QLQ-C30 global health status and all five functional subscales improved progressively in the observation group, whereas the control group showed minimal change; the largest effect was observed for emotional functioning (η^2^ = 0.340) (Table 2; Figure 3).

**Figure 4:**
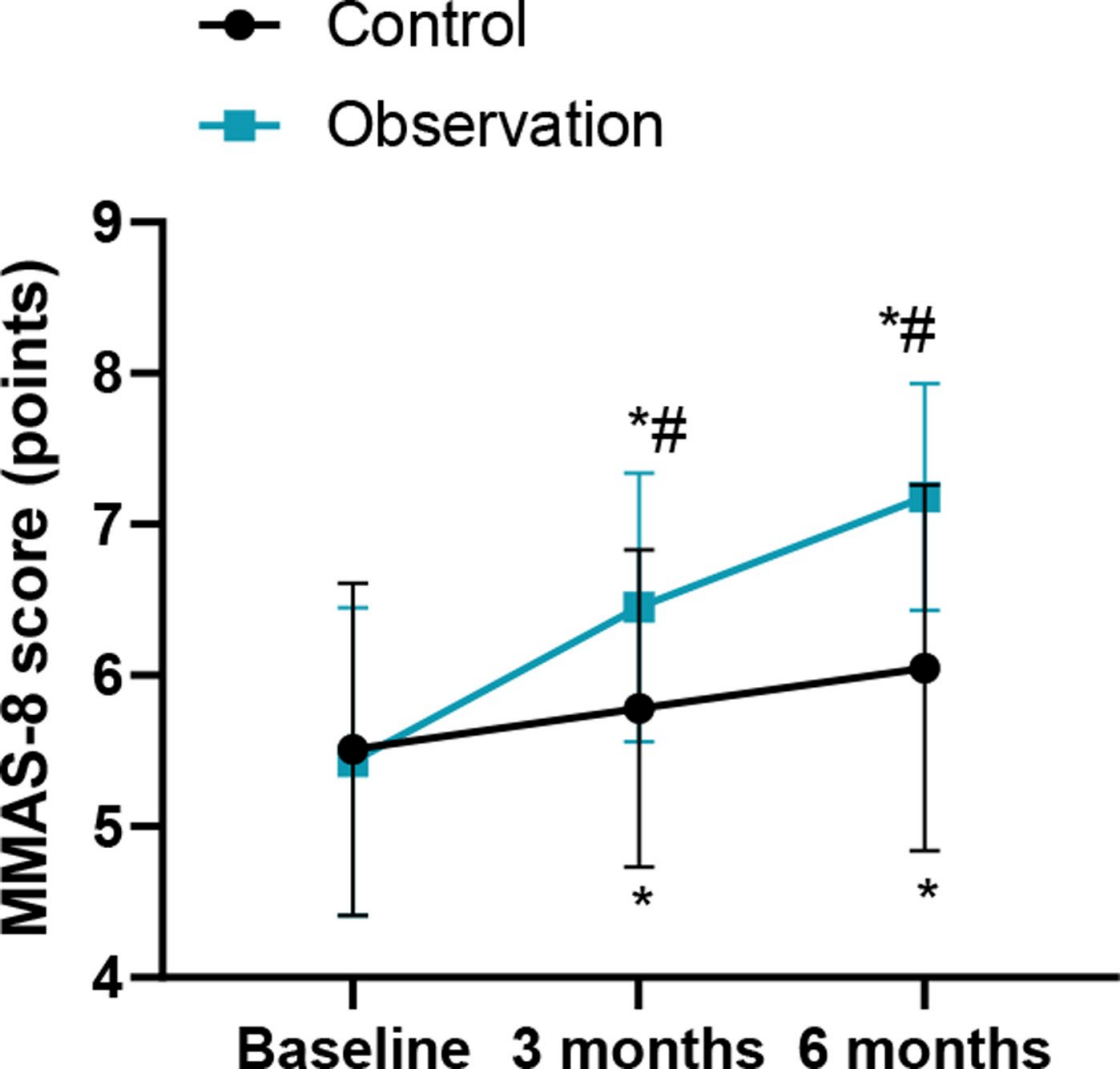
Medication adherence between the two groups. ^*^p < 0.05, compared with baseline; ^#^*p* < 0.05, compared with control group.

Medication adherence (MMAS-8) rose significantly in the observation group, reaching the “adequate adherence” threshold (≥6) by 6 months, while the control group remained borderline (η^2^ = 0.265; Table 2; Figure 4).

**Figure 5:**
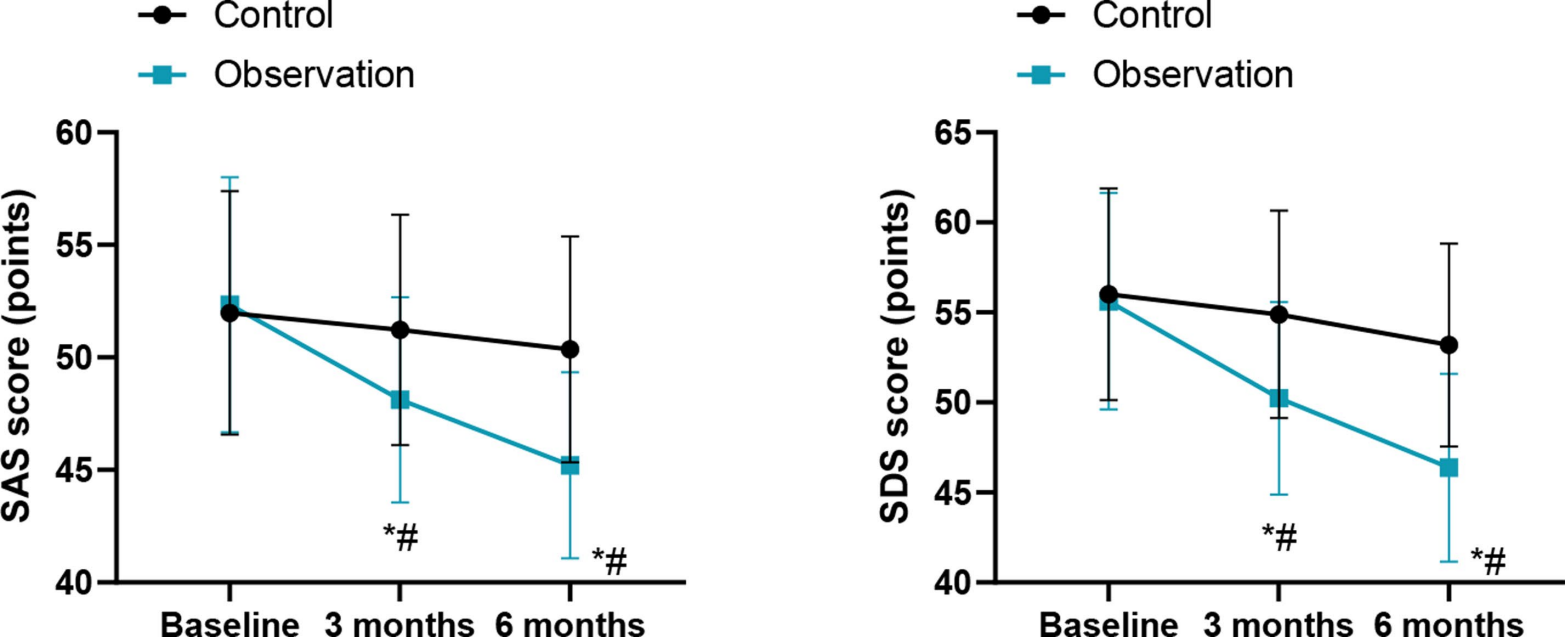
Anxiety and depression between the two groups. ^*^p < 0.05, compared with baseline; ^#^*p* < 0.05, compared with control group.

Anxiety (SAS) and depression (SDS) scores declined markedly in the observation group, falling below the clinical cutoff values (SAS < 50, SDS < 53) at 6 months; control group changes were minimal (SAS η^2^ = 0.298; SDS η^2^ = 0.309; Table 2; Figure 5).

**Figure 6:**
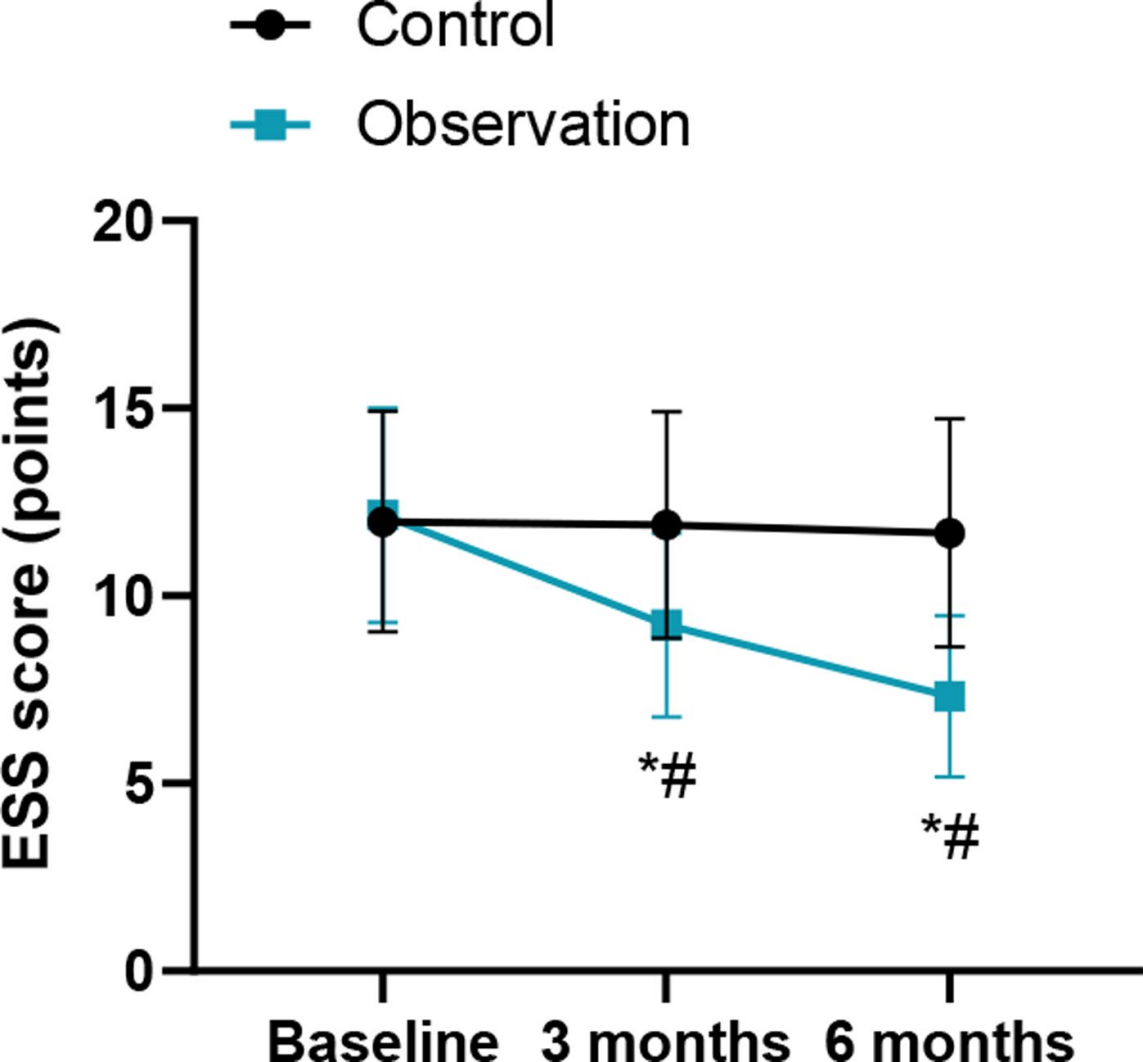
Daytime sleepiness between the two groups. ^*^p < 0.05, compared with baseline; ^#^*p* < 0.05, compared with control group.

Daytime sleepiness (ESS) decreased from the moderate excessive sleepiness range into the normal range in the observation group, whereas the control group remained essentially unchanged (η^2^ = 0.361; Table 2; Figure 6).

**Figure 7:**
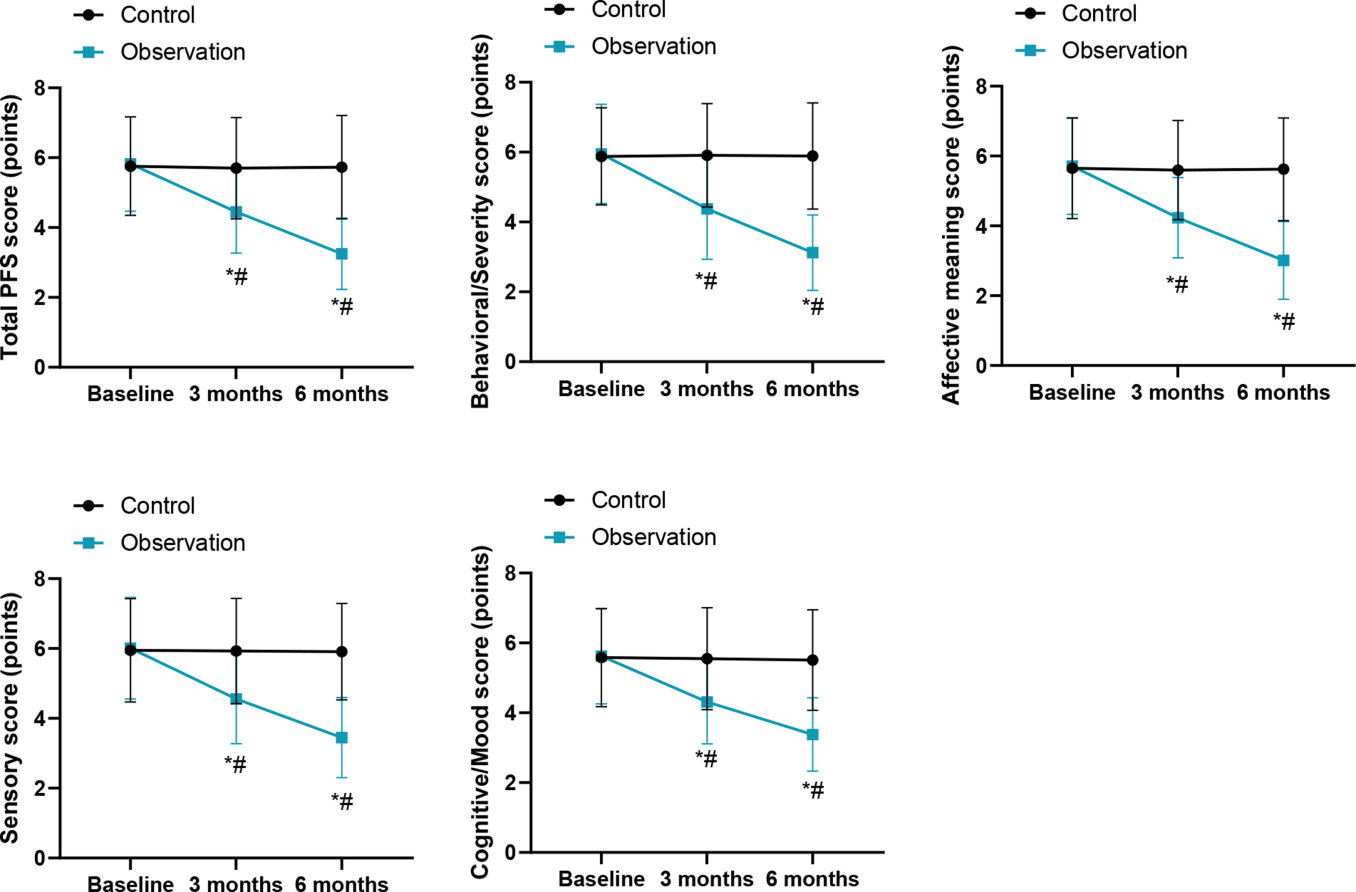
Cancer-related fatigue between the two groups. ^*^p < 0.05, compared with baseline; ^#^*p* < 0.05, compared with control group.

Cancer-related fatigue (PFS total and all subscales) improved substantially in the observation group, with the sensory subscale showing the largest effect (η^2^ = 0.392); the control group showed no significant change (Table 2; Figure 7).

Descriptive stratified analyses by leukemia subtype (AML and ALL) showed consistent intervention effects across both subgroups, with all primary and secondary outcome trends concordant with the overall cohort results (Supplementary Table S2). Formal statistical testing of subgroup interactions was not performed, as the study was not powered for subtype comparisons.

### Complications and rehospitalization

During the 6-month intervention period, the observation group had a significantly lower rate of any complication (11.11% vs. 29.63%, *p* = 0.016) and a lower rate of unplanned rehospitalization (7.41% vs. 24.07%, *p* = 0.012) compared with the control group (Table 3). Stratified analyses by leukemia subtype showed a consistent direction of effect for reduced complication and rehospitalization rates in both the AML and ALL subgroups; however, statistical significance was attenuated in the smaller ALL cohort due to limited sample size (Supplementary Table S2).

**Table 3:** Complications and rehospitalization between the two groups.

Complication	Observation (*n* = 54)	Control (*n* = 54)	*p*
Infection	4 (7.41%)	10 (18.52%)	0.084
Bleeding	1 (1.85%)	3 (5.56%)	0.308
Oral mucositis (Grade ≥2)	1 (1.85%)	3 (5.56%)	0.308
Any complication	6 (11.11%)	16 (29.63%)	0.016
Unplanned rehospitalization	4 (7.41%)	13 (24.07%)	6.31

## Discussion

This single-center randomized controlled trial provides robust evidence that a 6-month mHealth-supported continuous care program yields multifaceted benefits for AL patients in post-discharge remission periods. Rather than stemming from any single functional module, the clinical gains arise from the synergistic integration of real-time monitoring, tiered alert triage, targeted education, and on-demand support, which collectively transform post-discharge care from passive, scheduled follow-up into proactive, risk-stratified continuous surveillance.

The substantial improvement in self-management behaviors is likely attributable to the platform’s ability to translate abstract self-care advice into structured daily routines. The daily health diary fostered a habit of systematic self-observation, while automated alerts provided immediate, actionable feedback that reinforced recognition of critical signs. Combined with iterative educational content and easy access to nurse consultation, this “learning by doing” approach empowered patients to become active participants in their own care. For AL patients, who face high risks of acute complications during home-based myelosuppression periods, improved self-management ability is particularly clinically meaningful—it enables earlier identification of danger signals and reduces the risk of delayed medical attention. These findings are consistent with meta-analytic evidence from general oncology populations, while filling the evidence gap specifically for the AL cohort (23).

Quality of life gains exceeded the minimal clinically important difference and were evident across all functional domains, with the largest effect observed in emotional functioning. The mechanisms are likely multifactorial: earlier control of infection and bleeding complications reduced physical symptom burden, which in turn improved physical and role functioning; psychological support components, including on-demand video consultations and a moderated peer forum, mitigated emotional distress and social isolation; and fewer rehospitalizations allowed patients to remain in their home environment. These results align with prior reports that mHealth interventions can reduce psychological distress and improve QoL in oncology settings (24, 25).

Medication adherence reached the adequate threshold in the observation group, driven by the integrated combination of automated push notifications and a nurse backup system for missed doses. This dual approach addressed both unintentional forgetfulness and intentional non-adherence due to concern about adverse effects, which is particularly important for AL patients receiving complex multi-drug maintenance regimens with strict timing requirements. The findings are consistent with prior evidence supporting digital interventions for medication adherence in hematologic diseases.

The observed improvements in anxiety, depression, daytime sleepiness, and cancer-related fatigue appear to be interrelated components of a broader therapeutic effect. For AL patients in remission, persistent worry about febrile neutropenia and unexpected bleeding is a core source of psychological stress; the platform’s 30-min rapid response mechanism for abnormal alerts provided a tangible psychological “safety net” that reduced uncertainty and hypervigilance, which in turn alleviated anxiety and improved nighttime sleep quality. On-demand psychological support and peer interaction further normalized distress and fostered adaptive coping, in line with the known benefits of e-Health interventions for mitigating psychological distress in hematologic malignancies (26). Physiologically, better disease control through early detection of complications, combined with nutritional and sleep hygiene guidance, likely contributed to the large reductions in fatigue and sleepiness, forming a positive feedback loop between physical and mental health.

Clinically, the intervention yielded marked relative reductions in any complication and unplanned rehospitalization. These hard clinical endpoints support the core value of the automated early warning system: real-time capture of fever, bleeding, or other warning signs during the myelosuppression window enabled rapid nursing triage and early medical intervention, likely preventing progression to severe sepsis or life-threatening hemorrhage. This risk-stratified, responsive follow-up model represents a meaningful advance over traditional fixed-schedule telephone contact, which often misses the optimal intervention window for rapidly progressing AL-related complications.

The inclusion of both AML and ALL patients better reflects real-world clinical practice. Descriptive stratified analyses showed that the intervention effects were directionally consistent across both subtypes, with no obvious heterogeneity in efficacy trends (Supplementary Table S2), although statistical power was insufficient for formal interaction testing. Future adequately powered multicenter studies are needed to determine whether subtype-specific adaptations (e.g., more intensive monitoring during intensive consolidation phases for AML) can further optimize outcomes.

This study has several notable limitations that should be considered when interpreting the findings. First, the single-center design and the requirement for smartphone ownership and WeChat literacy limit generalizability. Notably, elderly patients with low digital literacy, who are at the highest risk of adverse home events during myelosuppression, were underrepresented in this cohort; whether the intervention can be adapted for this vulnerable group (e.g., with caregiver-assisted operation) remains to be explored. In addition, treatment protocols and supportive care strategies were relatively homogeneous at our center, and the generalizability to institutions with different maintenance chemotherapy regimens requires further verification. Second, blinding of participants and intervention nurses was not feasible, which may introduce performance bias; however, objective outcomes such as rehospitalization are less susceptible. Third, the 6-month follow-up cannot establish the long-term sustainability of benefits beyond the intervention period. Fourth, objective measures of sleep and fatigue (e.g., actigraphy or biomarkers) were not employed. Fifth, no cost-effectiveness analysis was conducted. Sixth, patients with severe comorbidities or short life expectancy were excluded. Despite these limitations, the consistent improvements across all measured domains, high patient adherence to the platform, and significant reductions in hard clinical endpoints support the feasibility and clinical potential of this mHealth continuous care model, pending further validation in multicenter, diverse populations.

## Conclusion

In summary, this randomized controlled trial demonstrates that a 6-month mHealth-supported continuous care program delivers medium-to-large, sustained improvements in self-management, quality of life, medication adherence, psychological status, sleepiness, and cancer-related fatigue, while concurrently reducing complications and unplanned rehospitalizations in AL patients in post-discharge remission. These single-center findings provide preliminary evidence supporting the clinical utility of mHealth-based continuous care for AL home management. However, given the digital literacy requirements and single-center study context, multicenter trials with more diverse populations, longer follow-up, and formal economic evaluation are warranted before widespread clinical implementation.

## Data Availability

The original contributions presented in the study are included in the article/Supplementary material, further inquiries can be directed to the corresponding author.
