# Supplementary material for: Effects of continuous care based on a mobile health platform on self-management behaviors and quality of life in patients with acute leukemia
Source: Front Med (Lausanne). 2026 Jul 22;13:1880270. doi: 10.3389/fmed.2026.1880270 (PMC13437363; doi:10.3389/fmed.2026.1880270)
Supplement: Supplementary file 1 [file Table_1.DOCX]

**Table S1 Baseline characteristics stratified by leukemia subtype**

| Characteristic | AML (n=78) | | | ALL (n=30) | | |
| --- | --- | --- | --- | --- | --- | --- |
|  | Observation (n=38) | Control (n=40) | P-value | Observation (n=16) | Control (n=14) | P-value |
| **Age (years), mean ± SD** | 49.2 ± 12.1 | 50.3 ± 12.8 | 0.698 | 39.8 ± 11.5 | 38.9 ± 12.1 | 0.835 |
| Age ≥60 years, n (%) | 8 (21.1) | 10 (25.0) | 0.678 | 1 (6.3) | 1 (7.1) | 0.923 |
| **Sex, n (%)** |  |  | 0.647 |  |  | 0.966 |
| Male | 22 (57.9) | 21 (52.5) |  | 9 (56.3) | 8 (57.1) |  |
| Female | 16 (42.1) | 19 (47.5) |  | 7 (43.8) | 6 (42.9) |  |
| **KPS score, mean ± SD** | 71.8 ± 8.9 | 71.2 ± 9.4 | 0.773 | 74.1 ± 7.8 | 73.9 ± 8.2 | 0.946 |
| **WBC (×10⁹/L), mean ± SD** | 32.4 ± 19.2 | 34.1 ± 20.8 | 0.708 | 19.8 ± 12.4 | 18.6 ± 13.1 | 0.798 |
| **Hemoglobin (g/L), mean ± SD** | 87.2 ± 15.8 | 85.4 ± 17.2 | 0.632 | 94.6 ± 13.2 | 93.8 ± 14.5 | 0.876 |
| **Platelet (×10⁹/L), mean ± SD** | 48.6 ± 26.4 | 51.2 ± 28.6 | 0.681 | 61.2 ± 32.1 | 66.4 ± 34.2 | 0.676 |
| **Cytogenetic risk, n (%)** |  |  | 0.889 |  |  | 0.812 |
| Favorable | 6 (15.8) | 8 (20.0) |  | 4 (25.0) | 4 (28.6) |  |
| Intermediate | 20 (52.6) | 18 (45.0) |  | 8 (50.0) | 8 (57.1) |  |
| Adverse | 12 (31.6) | 14 (35.0) |  | 4 (25.0) | 2 (14.3) |  |
| **Received allo-HSCT, n (%)** | 8 (21.1) | 10 (25.0) | 0.679 | 4 (25.0) | 4 (28.6) | 0.825 |

**Table S2 Main outcomes stratified by leukemia subtype at 6 months**

| Outcome | Time | AML (n = 78) | | | ALL (n = 30) | | |
| --- | --- | --- | --- | --- | --- | --- | --- |
|  |  | Obs (n = 38) | Con (n = 40) | P | Obs (n = 16) | Con (n = 14) | P |
| **SUPPH total** | Baseline | 55.4 ± 7.3 | 55.8 ± 7.6 | 0.812 | 56.3 ± 7.1 | 56.6 ± 7.4 | 0.910 |
|  | 6 months | 85.8 ± 6.4 | 70.6 ± 8.8 | <0.001 | 87.9 ± 5.4 | 73.1 ± 7.9 | <0.001 |
| **QLQ-C30 global** | Baseline | 58.2 ± 9.3 | 59.0 ± 9.6 | 0.711 | 59.0 ± 9.1 | 59.5 ± 9.4 | 0.884 |
|  | 6 months | 77.6 ± 7.5 | 61.8 ± 9.4 | <0.001 | 80.2 ± 6.2 | 64.3 ± 8.5 | <0.001 |
| **MMAS-8** | Baseline | 5.40 ± 1.03 | 5.55 ± 1.12 | 0.539 | 5.50 ± 1.00 | 5.65 ± 1.05 | 0.697 |
|  | 6 months | 7.12 ± 0.78 | 5.98 ± 1.24 | <0.001 | 7.32 ± 0.68 | 6.25 ± 1.12 | 0.003 |
| **SAS** | Baseline | 52.5 ± 5.7 | 52.0 ± 5.5 | 0.694 | 52.0 ± 5.6 | 51.6 ± 5.4 | 0.846 |
|  | 6 months | 45.6 ± 4.2 | 50.8 ± 5.1 | <0.001 | 44.2 ± 3.9 | 49.1 ± 4.8 | 0.004 |
| **SDS** | Baseline | 55.8 ± 6.1 | 55.2 ± 5.9 | 0.658 | 55.2 ± 5.8 | 54.5 ± 5.8 | 0.744 |
|  | 6 months | 46.9 ± 5.3 | 53.6 ± 5.7 | <0.001 | 45.2 ± 4.9 | 52.1 ± 5.4 | 0.001 |
| **ESS** | Baseline | 12.2 ± 2.9 | 12.0 ± 3.0 | 0.765 | 12.0 ± 2.8 | 11.9 ± 2.9 | 0.925 |
|  | 6 months | 7.5 ± 2.2 | 11.8 ± 3.1 | <0.001 | 6.9 ± 1.9 | 11.2 ± 2.8 | <0.001 |
| **PFS total** | Baseline | 5.85 ± 1.36 | 5.80 ± 1.42 | 0.873 | 5.75 ± 1.33 | 5.65 ± 1.39 | 0.843 |
|  | 6 months | 3.31 ± 1.05 | 5.78 ± 1.52 | <0.001 | 3.08 ± 0.94 | 5.58 ± 1.38 | <0.001 |
| **Any complication, n (%)** | 6 months | 5 (13.2) | 12 (30.0) | 0.062 | 1 (6.3) | 4 (28.6) | 0.142 |
| **Rehospitalization, n (%)** | 6 months | 3 (7.9) | 10 (25.0) | 0.039 | 1 (6.3) | 3 (21.4) | 0.286 |
